# Critical Care Cardiology: Embracing its Past for a Brighter Future

**DOI:** 10.1016/j.jacadv.2025.101816

**Published:** 2025-05-28

**Authors:** Barinder S. Hansra, Andrea M. Elliott, Ann Gage, Christopher F. Barnett, Brandon Wiley

**Affiliations:** a Division of Cardiology, Vanderbilt University Medical Center, Nashville, Tennessee, USA; b Division of Cardiology, University of Minnesota Medical Center, Minneapolis, Minnesota, USA; c Division of Cardiology, TriStar Centennial Medical Center, Nashville, Tennessee, USA; d Division of Cardiology, University of California Medical Center, San Francisco, San Francisco, California, USA; e Division of Cardiology, Los Angeles County Medical Center, Los Angeles, California, USA

**Keywords:** cardiology, critical care cardiology, leadership

## Historical perspective

In the United States, Dr Max Weil, a cardiologist by training, is often credited as a “father of modern-day critical care medicine (CCM).” In the early 1960s, he established a four-bed shock ward at Los Angeles County/University of Southern California Medical Center and made key contributions such as computerized patient monitoring, blood lactate measurement for tissue perfusion, and “stat labs.” In the 1970s, Dr Weil, along with 25 other specialists, founded the Society of Critical Care Medicine.

Extensive evidence supports the benefits of intensivist-led staffing models in improving intensive care unit (ICU) patient outcomes, reducing morbidity and mortality and lowering costs of care.^1^ The benefits apply to both pediatric and adult ICUs across different settings.^1^ Effective leadership of the multidisciplinary team, a key skill acquired during CCM training, is a primary driver of improved outcomes in ICU patients.^1^

Conversely, there are differing viewpoints regarding the optimal critical care cardiology (CCC) training and a topic of ongoing discussion among professional cardiovascular societies. For instance, trainees pursuing advanced heart failure and transplant cardiology (AHFTC) also have a strong interest in CCC.^2^ Proposals to introduce a *distinction in critical care cardiology* by incorporating a pathway in the AHFTC training year with focused skills and experiences commonly encountered in the cardiac intensive care unit (CICU).^2^ Challenges to this model include: eligibility for American Board of Internal Medicine (ABIM) certification, this model is only feasible at large quaternary centers with established AHFTC training, and widespread acceptance of standardized metrics must be established.

## Coronary care units to cardiac intensive care units: a paradigm shift

Coronary care units (CCUs) originated in the 1960s with the goal of rapidly resuscitating patients with dysrhythmias complicating acute myocardial infarction (AMI). Over time, some CCUs at large medical centers in the United States and Canada evolved into in CICUs providing comprehensive critical care for patients with cardiovascular disease, spanning malignant arrhythmias to hemodynamically compromising valve disease to cardiac arrest.^3^ The rich history of CCM has intertwined with cardiology, and the value of a dedicated intensivist is associated with improvements in CICU care.^4^ We believe this concept can be expanded to critical care–trained cardiologists caring for complex patients in the modern CICU, to provide simultaneous cardiology and CCM and reduce the number of personnel needed to round on these patients.

Currently, the cardiology community is actively promoting and recruiting CCCs, to take care of the modern day CICU patient. A few examples of core competencies of CCM training, often not fully obtained in cardiology fellowships include: sepsis management, airway management including endotracheal intubation, management of surgical complications of cardiovascular disease, advanced ventilator management, management of multisystem organ failure, palliative critical care, and postoperative cardiac surgery management. Procedural competencies in bronchoscopy, endotracheal intubation, and tube thoracostomy are not taught in general cardiology fellowships. The burgeoning subspecialty of CCC aims to combine the core competencies of CCM and those of cardiology training to ensure the consistent evidence-based care is delivered to critically ill patients in the CICU.

## Current state-of-critical care cardiology

CCM became an internal medicine subspecialty over 40 years ago, with the first board examination and certificates issued by the ABIM in 1987. Successful CCM training necessitates mastery of both procedural skills and cognitive skills. The fundamental principle of CCC emphasizes that comprehensive care of the contemporary CICU patient requires the core competencies obtained in both cardiology and CCM training.

Central to CCM training is expertise in the management of noncardiac disease in medical, neurological, trauma, and surgical settings. These skillsets enhance the ability of CCC to deliver well-rounded and comprehensive care in the CICU. We are not only cardiologists but intensivists who have invested considerable time, effort, and energy into honing our critical care knowledge, familiarity with the best practices, and the body of literature for management of critically ill patients (Table 1).

**Table 1:** Meetings, Research Groups, Research Networks, and Key Journals With a Strong Emphasis in Critical Care Cardiology

Category	Name	Description/Focus	Frequency	Relevance
Meetings	NYU Cardiovascular Critical Care Symposium	Annual meeting focusing on cardiovascular critical care, with emphasis on advances in CICU management and resuscitation.	Annual	Cardiovascular critical care, ICU management, and emerging therapies
	MN Critical Care Cardiology Education Summit	Annual meeting focusing on education, innovation, and practical clinical practice guidance.	Annual	Cardiovascular critical care, medical education.
	UCARS (U.S. Cardiac Arrest Registry to Enhance Survival)	Collaborative research meeting focusing on outcomes and interventions for patients with cardiac arrest.	Annual	Cardiac arrest, resuscitation, survival outcomes.
	Streams of Cardiology and ICU	Series of educational and clinical meetings focusing on the intersection of cardiology and critical care management.	Annually/Biannually	Cardiology in the ICU, heart failure, arrhythmias, and other critical conditions.
	Houston Shock Symposium	Annual meeting of world experts to share knowledge, discuss challenging cases, and advance knowledge to a broad audience.	Annual	Cardiogenic shock, ICU management, heart failure.
Interest groups	Critical Care WG of ACC	Workgroup within the American College of Cardiology focusing on critical care issues, research, and education in cardiology.	Ongoing/Annual	Critical care cardiology, education, policy, clinical practice
	AHA Resuscitation Science and Therapy	AHA’s focus on advancing science and research in resuscitation and critical care.	Ongoing/Annual	Cardiac arrest, resuscitation, postcardiac arrest syndrome, and related research.
	Society for Critical Care Cardiology	Focuses on advancing the practice of cardiology within critical care settings, through research, education, and advocacy	Ongoing/Annual	Critical care cardiology, heart failure, mechanical circulatory support, and advanced therapies
Research networks	CCCTN (Critical Care Cardiology Trials Network)	Collaborative research network focused on conducting high-impact clinical trials in critical care cardiology	Ongoing	Clinical trials, cardiology in the ICU, heart failure, arrhythmias, and shock management.
	CSWG (Cardiogenic Shock Working Group)	Focused on research and collaboration surrounding shock, including cardiogenic and septic in critical care.	Ongoing	Shock, circulatory failure, hemodynamic support, advanced therapies.
	SHOCK-OPS (SHOCK-Outcome Prognostic Studies)	Research network studying outcomes and prognostics in patients with shock, particularly those with cardiovascular causes.	Ongoing	Shock, prognosis, outcomes, and treatment in cardiovascular critical care.
Key journals	*EHJ-ACC (European Heart Journal-Acute Cardiovascular Care)*	Journal focusing on acute cardiovascular conditions, including cardiology and critical care in the acute settings.	Monthly/Quarterly	Acute cardiovascular care, emergency cardiology, resuscitation, and critical care cardiology.
	*JACC Advances*	Journal dedicated to providing advances in cardiology with a focus on translation research and clinical practice.	Quarterly	Cardiology, with growing emphasis on cardiovascular critical care, ICU management, and innovations.
	*Journal of the American College of Cardiology (JACC)*	Leading cardiology journal, publishing high-impact studies on cardiovascular disease, including critical care topics.	Weekly	Cardiovascular research, clinical guidelines, cardiovascular disease, and management in critical care settings.
	*Circulation: Heart Failure*	Journal focused on heart failure and its management, with specific focus on acute and critical care settings.	Monthly	Acute heart failure, heart transplant, mechanical circulatory support, and ICU management.

ACC = American College of Cardiology; AHA = American Heart Association; CICU = cardiac intensive care unit; ICU = intensive care unit; MN = Minnesota; NYU = New York University; WG = Working Group.

An alternative perspective is that while noncritical care–trained cardiologist may not possess all the expertise required for managing every aspect of critical illness in the CICU, their specialized knowledge in cardiology, combined with their ability to work in a team-based care model, makes them valuable contributors to the CICU team. When supported by intensivists, these providers make a significant positive impact on the care of critically ill cardiac patients.

The growing demand for dedicated critical care cardiologists is evident through the increasing number of CCM programs offering training pathways for critical care cardiology and major institutions shifting their preferred staffing models to CICUs staffed and led by critical care cardiologists, dual-trained in both cardiology and CCM.^5^ These specialists offer full-spectrum cardiac and critical care but are uniquely positioned to offer specialized services such as transesophageal echo, bedside Swan-Ganz catheter placement/interpretation, optimization of ventilatory settings based on heart-lung interaction, and management of mechanical support (MCS).

## Historic trends in the CICU population

Before the establishment of CCUs in the 1960s, patients who suffered from AMI were primarily managed with bed rest and sedation; with associated in-hospital mortality of 30% and 1-year all-cause mortality of 45%.^3^ Advances in therapies have reduced mortality from AMI over the years from 20% to 25% in the 1980s to 10% to 15% in the 1990s to approximately 6% at present.^6^ Despite tremendous improvement in AMI pathology, mortality from cardiogenic shock (CS) remains 40% in contemporary populations.^7^ The high mortality of AMI lead to tireless work and the efforts to improve patient outcomes, similarly, the unacceptably high mortality of CS and other CCC illnesses requires reform and continued attention.

The CICU is home to a diverse patient population influenced by factors such as an aging demographic, acute/chronic sequelae of nonfatal MI, increasing rates of obesity/metabolic syndrome and complications of intravascular and implantable devices, all increasing patient susceptibility to critical illness and fundamentally altering the demographic present in the modern CICU.^3^ Furthermore, acute/chronic heart failure requiring temporary MCS, durable left ventricular assist device, or those who have undergone orthotopic heart transplantation add complexity to CICU management and underscore the need for specialized expertise. The prevalence of noncardiovascular diagnoses present in the CICU has increased, with the rate greatest for comorbid critical illnesses, including sepsis, acute kidney injury and respiratory failure, and increased use of mechanical ventilation, all which influence patient outcomes.^3^

In a national-level retrospective study of 3.4 million acute care hospitalizations with CICU stays from 2003 to 2013, demonstrated a significant rise in primary noncardiac diagnoses, surging from 38% to 51.7% between 2003 and 2013 among CICU admissions. Among these noncardiac diagnoses, infectious diseases and respiratory diseases increased from 7.8% to 15.1% and 6.0% to 7.8%, respectively.^8^ Patients exhibited increased use of hemodialysis, transfusions, and mechanical/noninvasive ventilation, alongside higher unadjusted and risk-adjusted in-hospital mortality. Consequently, the CICU is now characterized by a heterogeneous patient population resembling general medical ICUs who additionally, have may have unique additional cardiac-specific needs.^8^

Arguably, one of the most daunting disease processes encountered in the CICU is CS due to heterogeneity in underlying etiology, clinical manifestations, severity, and outcomes.^9^ Shock in all its forms is increasingly prevalent in the CICU and is associated with high mortality. CS secondary to AMI is declining, while CS from acute/chronic decompensated heart failure is on the rise. Patients experiencing mixed cardiogenic-septic shock represent a high-risk subgroup characterized by greater illness severity and worse outcomes.^9^ The simultaneous rise in the utilization of advanced MCS and associated complications (ischemic/hemorrhagic stroke, massive hemorrhage with transfusion protocols, acute limb ischemia, etc, are learned in dedicated CCM training) highlights the need for management by individuals with formalized CCM and cardiology training. In conjunction with the ABIM, American College of Cardiology Core Cardiology Training Statement and European Society of Cardiology aligns with at least 12 months of critical care time to be considered adequate training.^10^ While we recognize the value of additional 12 months of dedicated critical care training for cardiologist, there are barriers to this extending training period, including: additional costs associated with applications and interviews, prolonged duration of training, and the potential need for relocation. These factors may discourage motivated and qualified trainees from pursuing this path. Therefore, it is essential to continuously assess best practices in cardiology fellowship programs to address these obstacles and ensure aspiring cardiologists can pursue specialized training in critical care without unnecessary burdens.

## The future of critical care cardiology

Wayne Gretzky remarked, “skate to where the puck is going, not where it has been,” a poignant metaphor for CCC. Our field stands at a pivotal juncture, reminiscent of past crossroads. It is now time to formalize training, develop professional societies, establish core competencies, and develop metrics for assessment.

The era of CICUs isolating themselves from the critical care community must come to an end; instead, we must forge connections and become collaborative partners. Understanding CCM makes us better in our role as critical care cardiologists and ultimately will lead to tangible progress for patients with improvements in mortality and complications. Furthermore, increasing collaborations with broader groups of CCM practitioners and researchers will accelerate science and innovation, education, and improve clinical care for cardiac and noncardiac critically ill patients.

Reflecting on the transformative journeys of other medical disciplines, we, as cardiac intensivists, advocate for a mandatory, comprehensive, and diverse year of critical care training for physicians intending to care for the heterogeneous patient population constituting the contemporary CICU. Let us, as a community, instead of skating to where the puck has been, skate together to where the puck is going to be.

## Funding support and author disclosures

The authors have reported that they have no relationships relevant to the contents of this paper to disclose.
